# Clinical characteristics and early admission predictors of in-hospital death in acute paraquat poisoning: a retrospective cohort study of 128 patients

**DOI:** 10.3389/fmed.2026.1815780

**Published:** 2026-05-13

**Authors:** Yuquan Chen, Meiwen Xie, Yifan Ye, Yuqiang Lin, Zhiqian Yang, Zhi Wang

**Affiliations:** Department of Occupational Diseases, Guangzhou Twelfth People's Hospital (Guangzhou Occupational Disease Prevention and Treatment Hospital), Guangzhou, China

**Keywords:** biomarkers, cohort studies, mortality, paraquat poisoning, prognosis, receiver operating characteristic curve

## Abstract

**Background:**

Acute paraquat (PQ) poisoning is associated with high mortality and rapid multi-organ injury. Early risk stratification is particularly important when plasma or urine PQ concentrations are unavailable. This study aimed to characterize acute PQ poisoning and identify routinely available admission variables associated with in-hospital death.

**Methods:**

We retrospectively enrolled 128 consecutive patients with acute PQ poisoning admitted to Guangzhou Twelfth People’s Hospital between January 2008 and December 2020. Patients were categorized as survivors (*n* = 46) or non-survivors (*n* = 82) according to in-hospital outcome. Continuous variables were analyzed using the Mann–Whitney U test and categorical variables using the chi-square test or Fisher’s exact test, as appropriate. Clinically relevant admission variables were screened for collinearity and entered into a backward stepwise binary logistic regression model in SPSS 25.0. ROC curves were generated in R, and AUCs with 95% confidence intervals were calculated.

**Results:**

The in-hospital mortality rate was 64.06%. Compared with survivors, non-survivors had significantly higher ingested dose, coagulation indices, inflammatory markers, liver injury markers, renal function indices, and myocardial injury markers (all *p* < 0.05). In multivariable analysis, ingested dose (OR = 1.016, 95% CI: 1.003–1.029, *p* = 0.015) and AST (OR = 1.027, 95% CI: 1.011–1.042, *p* = 0.001) were independently associated with in-hospital death. AST showed the best discrimination among single markers (AUC = 0.911, 95% CI: 0.855–0.958).

**Conclusion:**

Ingested dose and AST were independently associated with in-hospital death in acute PQ poisoning, and AST showed the best single-marker discriminative performance.

## 1 Introduction

Paraquat (PQ) is a rapidly acting non-selective herbicide with substantial human toxicity. Intentional or accidental ingestion can cause fulminant poisoning with a high case-fatality rate, particularly in Asia (1). Acute PQ poisoning is characterized by oxidative stress, inflammatory activation, and progressive multi-organ injury, with prominent involvement of the lungs, kidneys, and liver (2, 3). Although the use of PQ has been restricted or banned in many jurisdictions, severe poisoning continues to be encountered in clinical practice (4, 5).

Early risk stratification is essential because no antidote with proven efficacy is available and treatment remains largely supportive (2). Plasma PQ concentration and concentration-time nomograms are informative when available (6, 7), but these assays are unavailable in many hospitals, particularly in resource-limited settings (2, 8). Accordingly, admission variables derived from routine laboratory tests remain pragmatic candidates for early prognostic assessment. Earlier studies have evaluated APACHE II-based scores, arterial lactate, and simple admission laboratory markers as accessible alternatives when toxicokinetic testing is unavailable (9–14).

Previous studies have proposed a range of prognostic markers and prediction tools for PQ poisoning, including hematological indices and multivariable prediction models (13–17). However, some analyses included closely related biomarkers in the same model or did not clearly separate admission predictors from post-admission process variables (18, 19). In contrast to approaches that combine multiple overlapping indicators, the present study focused on routinely available first-day variables and sought a more parsimonious early-admission model after collinearity-oriented screening. In this retrospective cohort of 128 consecutive patients, we therefore re-evaluated routinely available first-day clinical variables using a prespecified non-parametric framework, collinearity-aware multivariable modeling, and ROC analysis, with in-hospital death as the primary endpoint.

## 2 Patients and methods

### 2.1 Study design and setting

This was a single-center retrospective cohort study conducted at Guangzhou Twelfth People’s Hospital, a referral center for occupational and toxicological diseases. We reviewed consecutive admissions for acute PQ poisoning from January 2008 to December 2020.

### 2.2 Participant selection

A total of 202 patients were initially screened. Diagnosis was based on a documented history of PQ exposure, compatible clinical manifestations, and toxicological evidence when available in the medical record (such as a qualitative toxicology result or a documented toxicology-based clinical diagnosis).

**Figure 1:**
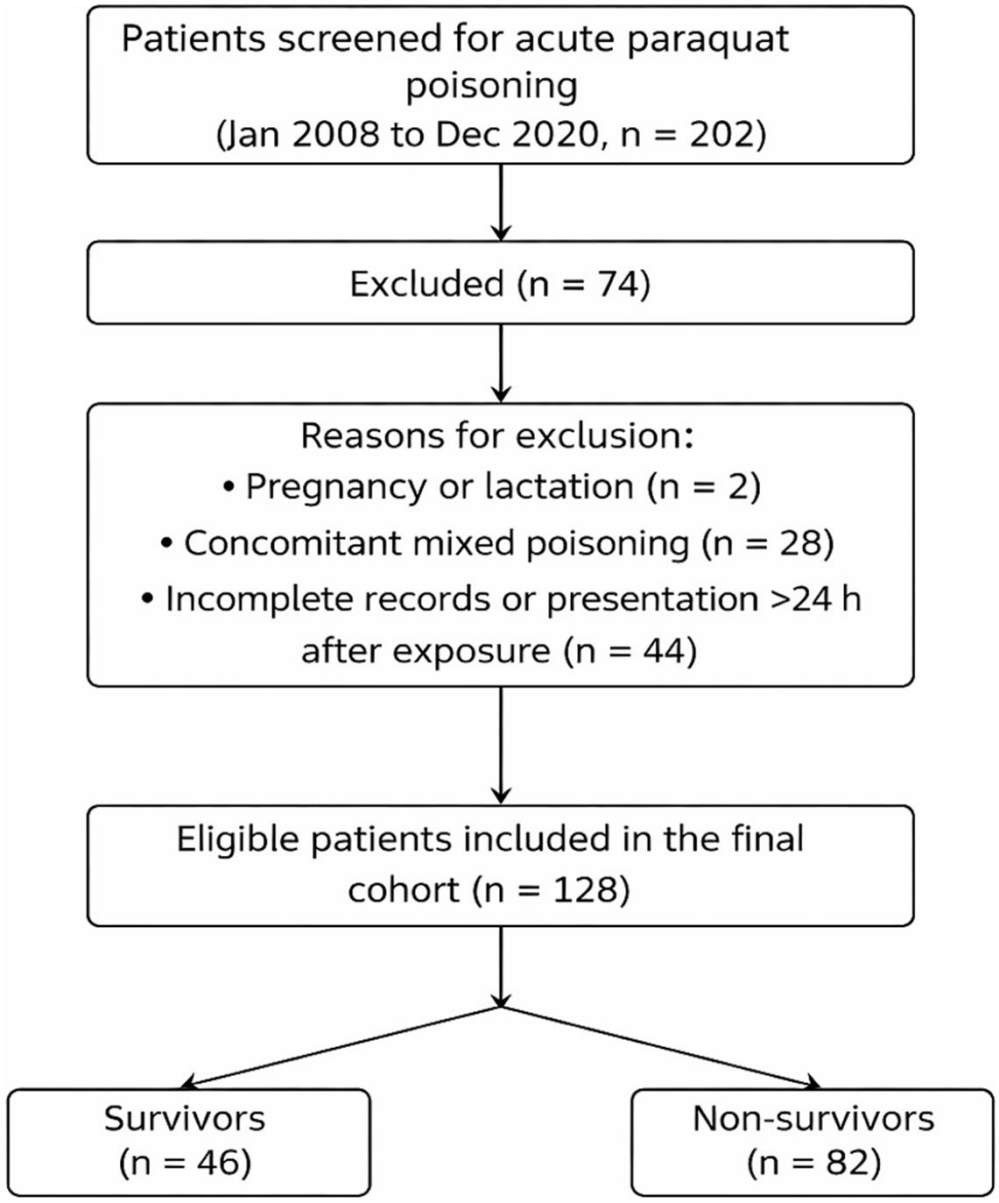
Flow diagram of patient selection.

Inclusion criteria were: (1) presentation within 24 h after exposure; (2) confirmed acute PQ poisoning; and (3) availability of first-day blood routine, biochemical, coagulation, and key clinical data. Exclusion criteria were: (1) concomitant poisoning with other toxic agents; (2) pregnancy or lactation; and (3) incomplete records for the predefined baseline variables or presentation later than 24 h after exposure. Patients with uncertain diagnosis were not retained in the screened cohort. After exclusion of 74 patients (2 pregnancy/lactation, 28 mixed poisoning, and 44 incomplete records or delayed presentation), 128 patients were included in the final analysis (Figure 1).

### 2.3 Data sources, measurements, and treatment overview

Demographic and clinical variables were abstracted retrospectively from electronic and archived medical records using a predefined data collection form. Extracted variables included sex, age, route of exposure, estimated ingested dose, gastric lavage status, length of hospital stay, and first-day laboratory indices. Ingested dose was recorded in milliliters according to the clinical history documented at admission, usually based on patient or family report. Because the exact concentration of the commercial formulation and toxicokinetic PQ measurements were not consistently available, ingested dose should be interpreted as an approximate clinical estimate rather than a chemical exposure measurement.

The first recorded laboratory values within 24 h after admission were used for analysis, including white blood cell count, liver function, renal function, coagulation indices, creatine kinase markers, LDH, and α-HBDH. Patients were managed according to institutional clinical practice during the study period, including gastrointestinal decontamination when appropriate, hemoperfusion or other extracorporeal support when indicated, organ support, and symptomatic treatment.

### 2.4 Outcome definition and missing data

The primary outcome was in-hospital death during the index admission. Survivors were patients discharged alive, and non-survivors were patients who died before discharge. Missing data were handled by screening-stage exclusion: patients with incomplete predefined baseline variables were excluded before analysis. Consequently, the final cohort had complete data for the variables entered into the regression model.

### 2.5 Statistical analysis

Statistical analysis was performed using SPSS 25.0. Normality of continuous variables was assessed using the Shapiro–Wilk test. Because continuous variables were non-normally distributed, they are presented as median (P_25_, P_75_), and between-group comparisons were performed using the Mann–Whitney U test. Categorical variables are presented as n (%) and were compared using the chi-square test or Fisher’s exact test, as appropriate. No formal *a priori* sample size calculation was performed because this was a retrospective study of all eligible consecutive cases during the study period; however, the final multivariable model was kept parsimonious relative to the number of outcome events.

Candidate variables for multivariable analysis were chosen according to clinical relevance, admission timing, and univariable signal. To reduce unstable estimates caused by closely related biomarkers, highly correlated variable pairs were not entered simultaneously. One clinically representative marker from each correlated pair was retained before modeling: WBC instead of NEU, PT-INR instead of PT, AST instead of ALT, TBIL instead of DBIL, creatinine instead of urea, CK-MB instead of CK, and LDH instead of α-HBDH. Variance inflation factors were then checked in the candidate set, and no problematic residual multicollinearity was found (all VIFs < 5). A backward stepwise binary logistic regression model was fitted, and a parsimonious final model retaining variables with *p* < 0.05 was reported. ROC curves were generated for the independent factors retained in the final model. Optimal thresholds were identified by the maximum Youden index. Because thresholds were derived from the same dataset, ROC cut-offs should be regarded as exploratory and require external validation. All tests were two-sided, and *p* < 0.05 was considered statistically significant.

### 2.6 Ethics statement

This retrospective study was approved by the Medical Ethics Committee of Guangzhou Occupational Disease Prevention and Treatment Hospital (Guangzhou Twelfth People’s Hospital). All medical records were reviewed in a de-identified manner, and access to study data was restricted to the investigators. Because this study used anonymized retrospective clinical data, the requirement for written informed consent was waived by the Medical Ethics Committee.

## 3 Results

### 3.1 Study flow and baseline characteristics

A total of 202 patients with acute PQ poisoning were screened and 128 met the predefined eligibility criteria (Figure 1). Among the included patients, 46 survived and 82 died during hospitalization, corresponding to an in-hospital mortality rate of 64.06%. The overall median age was 27.5 years, and 59 patients (46.1%) were male.

No significant between-group differences were found for age, sex, or gastric lavage status (all *p* > 0.05). In contrast, the estimated ingested dose was markedly higher in non-survivors than in survivors (*p* < 0.001). Baseline characteristics are summarized in Table 1.

**Table 1:** Baseline characteristics of patients with acute paraquat poisoning.

Variable	All patients (*n* = 128)	Survivor group (*n* = 46)	Non-survivor group (*n* = 82)	*Z/χ* ^2^	*p* value
Age (years)	27.5 (20.0, 38.0)	29.0 (19.3, 36.0)	26.5 (21.0, 38.0)	−0.075	0.943
Sex (male/female)	59/69	16/30	43/39	3.697	0.055
Ingested dose (mL)	31.5 (15.0, 70.0)	15.5 (10.0, 30.0)	50.0 (30.0, 100.0)	−5.548	<0.001
Gastric lavage (yes/no)	112/16	40/6	72/10	0.019	0.889

Continuous data are presented as median (P_25_, P_75_); categorical data are presented as *n* or *n* (%).

### 3.2 Comparison of first-day laboratory parameters

Compared with survivors, non-survivors had significantly higher first-day values of WBC, NEU, ALT, AST, TBIL, creatinine, urea, PT, PT-INR, APTT, CK, CK-MB, LDH, and α-HBDH (all *p* < 0.05). FbgC did not differ significantly between groups (*p* = 0.067). These findings indicate that early abnormalities in inflammatory, hepatic, renal, coagulation, and tissue-injury markers were more pronounced among patients who died during hospitalization (Table 2).

**Table 2:** Univariate analysis of first-day laboratory parameters.

Variable	Survivor group	Non-survivor group	*Z*	*P* value
WBC (10^9^/L)	13.12 (8.26, 17.02)	20.12 (16.73, 26.05)	−5.840	<0.001
NEU (10^9^/L)	11.37 (6.80, 14.89)	18.46 (15.10, 23.12)	−5.520	<0.001
ALT (U/L)	14.55 (10.62, 20.20)	56.85 (24.98, 185.15)	−6.439	<0.001
AST (U/L)	21.45 (16.38, 28.70)	125.85 (48.05, 286.3)	−7.702	<0.001
TBIL (μmol/L)	17.05 (11.50, 25.27)	28.75 (18.15, 58.08)	−3.965	<0.001
Cr (μmol/L)	93.60 (66.72, 123.75)	305.40 (178.57, 375.98)	−6.635	<0.001
Urea (mmol/L)	4.34 (3.30, 6.93)	8.38 (6.21, 11.81)	−5.418	<0.001
PT (s)	11.95 (11.20, 13.07)	13.40 (12.12, 16.43)	−3.894	<0.001
PT-INR	1.02 (0.97, 1.12)	1.16 (1.03, 1.40)	−3.999	<0.001
APTT (s)	26.60 (22.50, 32.43)	31.70 (26.00, 43.80)	−3.002	0.003
FbgC (g/L)	2.13 (1.78, 2.51)	2.33 (1.87, 2.91)	−1.833	0.067
CK (U/L)	141.00 (90.00, 224.75)	399.50 (182.25, 715.50)	−5.008	<0.001
CK-MB (U/L)	17.25 (12.62, 24.85)	40.00 (24.25, 62.88)	−5.853	<0.001
LDH (U/L)	194.50 (150.00, 251.25)	352.50 (260.75, 616.50)	−6.878	<0.001
α-HBDH (U/L)	153.50 (126.00, 192.50)	274.50 (215.00, 454.00)	−6.799	<0.001

WBC, white blood cell count; NEU, neutrophil count; ALT, alanine aminotransferase; AST, aspartate aminotransferase; TBIL, total bilirubin; Cr, creatinine; PT-INR, prothrombin time international normalized ratio; APTT, activated partial thromboplastin time; FbgC, fibrinogen; CK-MB, creatine kinase-MB; LDH, lactate dehydrogenase; α-HBDH, α-hydroxybutyrate dehydrogenase.

### 3.3 Multivariable logistic regression analysis

Before multivariable modeling, correlated laboratory variables were screened to reduce collinearity. WBC was retained instead of NEU, PT-INR instead of PT, AST instead of ALT, TBIL instead of DBIL, creatinine instead of urea, CK-MB instead of CK, and LDH instead of α-HBDH. After this prescreening, all candidate variables showed acceptable collinearity diagnostics (all VIFs < 5). Length of hospital stay was not entered into the model because it was considered a post-admission process variable rather than an admission predictor.

Backward stepwise binary logistic regression identified ingested dose and AST as independent factors associated with in-hospital death (Table 3). Both variables were positively associated with death.

**Table 3:** Multivariable logistic regression analysis of in-hospital death (final step).

Variable	*B*	*SE*	Wald *χ*^2^	*P* value	OR	95% CI
Ingested dose	0.0156	0.0064	5.931	0.015	1.016	1.003–1.029
AST	0.0262	0.0077	11.656	0.001	1.027	1.011–1.042
Constant	−1.7830	0.4281	17.349	<0.001	0.168	0.073–0.389

Death was coded as 1 and survival as 0. Candidate variables were screened for collinearity before backward stepwise modeling.

### 3.4 ROC curve analysis

**Figure 2:**
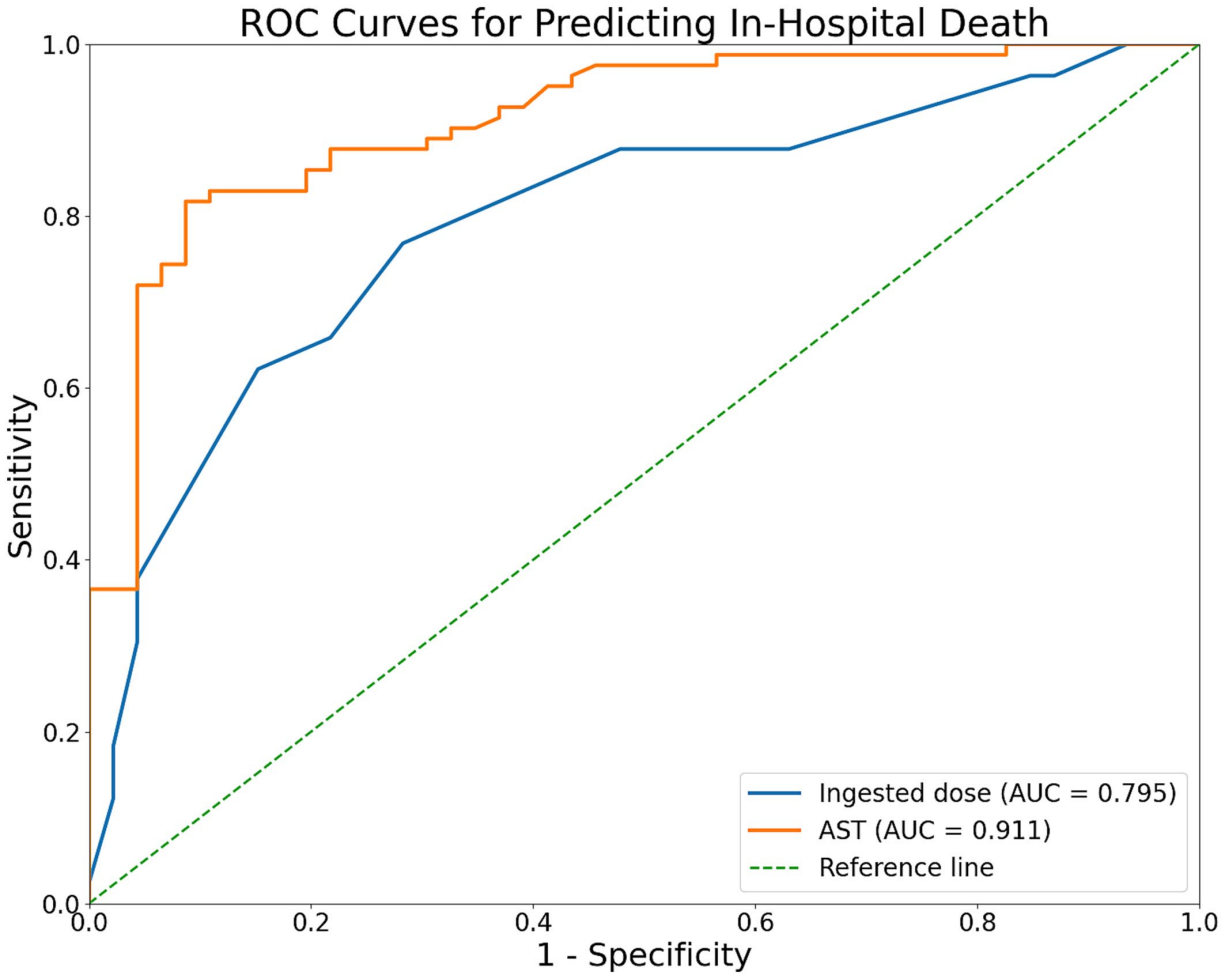
ROC curves of ingested dose and AST for predicting in-hospital death.

ROC curves were constructed for the two independent factors retained in the final model (Figure 2). AST showed the best individual discriminative performance, with an AUC of 0.911 (95% CI: 0.855–0.958), sensitivity of 81.7%, and specificity of 91.3%. The AUC for ingested dose was 0.795 (95% CI: 0.706–0.872; all *p* < 0.001). Because the thresholds were derived and tested in the same dataset, these cut-offs should be interpreted as exploratory (Table 4).

**Table 4:** ROC analysis of independent factors.

Variable	Youden index	Cut-off value	Sensitivity	Specificity	*P* value	AUC	95% CI
Ingested dose	0.486	30.00	0.768	0.717	<0.001	0.795	0.706–0.872
AST	0.730	40.80	0.817	0.913	<0.001	0.911	0.855–0.958

AUC, area under the receiver operating characteristic curve. Optimal cut-offs were defined by the maximum Youden index.

## 4 Discussion

Acute PQ poisoning remained highly lethal in this cohort, with an in-hospital mortality of 64.06%. This magnitude is consistent with previous cohorts of moderate-to-severe PQ ingestion and reinforces the continuing clinical value of early bedside risk stratification when quantitative toxicokinetic assays are unavailable (16, 17).

Estimated ingested dose remained a robust prognostic signal, consistent with studies linking higher exposure burden to mortality (17, 20). AST also retained an independent association with death after multivariable adjustment. At one level, this is biologically plausible because PQ toxicity can produce early hepatocellular injury as part of a broader systemic oxidative injury pattern, and recent human data suggest that liver injury occurring within 48 h is associated with mortality in PQ poisoning (3, 18, 19).

However, AST should not be interpreted only as a liver marker. AST is a cytosolic and mitochondrial enzyme distributed not only in the liver but also in myocardium and skeletal muscle, and it was historically used as a biomarker of myocardial injury (21, 22). In our cohort, the simultaneous elevation of CK-MB, LDH, and alpha-HBDH in non-survivors is consistent with this broader tissue-injury profile. Accordingly, the strong discriminatory performance of AST may reflect composite injury severity rather than isolated hepatic damage alone.

This interpretation is also compatible with the recognized cardiac toxicity of PQ. Experimental studies have shown that PQ can induce myocardial damage, contractile dysfunction, electrophysiologic abnormalities, and hemodynamic deterioration (23, 24). Clinical studies further suggest that cardiac involvement is prognostically relevant: QTc prolongation and other ECG abnormalities within 24 h have been associated with death or worse prognosis in acute PQ poisoning (25, 26). Therefore, the prognostic value of AST in our study may partly relate to PQ-associated myocardial injury and cardiovascular instability, especially in patients with more severe systemic poisoning.

Although several other laboratory markers-including LDH, creatinine, coagulation indices, and bilirubin-showed clear univariable associations with death, they did not remain in the final parsimonious model after collinearity-oriented screening and multivariable adjustment. Prior studies likewise support prognostic roles for renal dysfunction and kidney injury trajectories in PQ poisoning (20, 27, 28). Leukocyte-derived indices and arterial lactate have also shown prognostic value in other cohorts and meta-analyses (11, 29–31). This pattern supports the use of a simpler and more interpretable early-admission model, which may be more practical for bedside risk stratification than a larger panel of overlapping laboratory variables.

This study has several limitations. First, it was retrospective and single-center, which limits external validity. Second, the recruitment period was long (2008–2020); therefore, changes in referral patterns, case-mix, regulation of PQ, and treatment practice over time may have introduced temporal heterogeneity. Third, ingested dose was estimated from clinical history rather than systematically verified by toxicokinetic testing, and plasma or urine PQ concentrations were unavailable for most patients. Fourth, the exclusion of incomplete records may have introduced selection bias. Fifth, systematic cardiac biomarkers such as troponin and standardized ECG variables were not available for all patients, so the proposed link between AST and myocardial injury remains inferential rather than directly demonstrated in this cohort. Finally, although ROC thresholds and the final model showed good apparent discrimination, neither bootstrap-based internal validation nor external validation was performed. The present findings should therefore be regarded as clinically supportive and hypothesis-generating rather than definitive.

## 5 Conclusion

In this retrospective cohort of 128 patients with acute PQ poisoning, ingested dose and AST were independently associated with in-hospital death. Among the individual indicators, AST showed the best discriminative performance. Early assessment of these routinely available admission variables may assist bedside risk stratification, but external validation is required before broader prognostic use.

## Data Availability

The datasets analyzed during the current study are not publicly available due to privacy and ethical restrictions related to retrospective clinical records. Aggregated data supporting the conclusions are included in the article and Supplementary material. Further inquiries can be directed to the corresponding author.
